# Age-related alterations of brain network underlying the retrieval of emotional autobiographical memories: an fMRI study using independent component analysis

**DOI:** 10.3389/fnhum.2014.00629

**Published:** 2014-08-14

**Authors:** Ruiyang Ge, Yan Fu, Dahua Wang, Li Yao, Zhiying Long

**Affiliations:** ^1^State Key Laboratory of Cognitive Neuroscience and Learning and IDG/McGovern Institute for Brain Research, Beijing Normal UniversityBeijing, China; ^2^Center for Collaboration and Innovation in Brain and Learning Sciences, Beijing Normal UniversityBeijing, China; ^3^College of Information Science and Technology, Beijing Normal UniversityBeijing, China; ^4^College of Psychology, Beijing Normal UniversityBeijing, China; ^5^Action, Brain, and Cognition Laboratory and fMRIotago, Department of Psychology, University of OtagoDunedin, New Zealand

**Keywords:** emotional autobiographical memory, aging, positivity effect, fMRI, ICA

## Abstract

Normal aging has been shown to modulate the neural underpinnings of autobiographical memory and emotion processing. Moreover, previous researches have suggested that aging produces a “positivity effect” in autobiographical memory. Although a few imaging studies have investigated the neural mechanism of the positivity effect, the neural substrates underlying the positivity effect in emotional autobiographical memory is unclear. To understand the age-related neural changes in emotional autobiographical memory that underlie the positivity effect, the present functional magnetic resonance imaging (fMRI) study used the independent component analysis (ICA) method to compare brain networks in younger and older adults as they retrieved positive and negative autobiographical events. Compared to their younger counterparts, older adults reported relatively higher positive feelings when retrieving emotional autobiographical events. Imaging data indicated an age-related reversal within the ventromedial prefrontal/anterior cingulate cortex (VMPFC/ACC) and the left amygdala of the brain networks that were engaged in the retrieval of autobiographical events with different valence. The retrieval of negative events compared to positive events induced stronger activity in the VMPFC/ACC and weaker activity in the amygdala for the older adults, whereas the younger adults showed a reversed pattern. Moreover, activity in the VMPFC/ACC within the task-related networks showed a negative correlation with the emotional valence intensity. These results may suggest that the positivity effect in older adults' autobiographical memories is potentially due to age-related changes in controlled emotional processing implemented by the VMPFC/ACC-amygdala circuit.

## Introduction

Human aging is accompanied by the decline of various cognitive abilities and alterations in the function and structure of the neural circuits underlying sensorimotor, cognitive and emotion processing. A large body of evidence demonstrates that prominent changes occur across the human life span in autobiographical memory that is an extremely complex memory system (Brewer, 1986) with high personal significance and emotional valence.

Changes associated with aging in autobiographical memory have been extensively investigated. At the behavioral level, memories of past events generated by older adults contained less episodic detail and more generic content than those remembered by younger adults (Levine et al., 2002). In contrast to younger adults, older adults tended to report a stronger recollective experience (Janssen et al., 2011) and take relatively more time to gain access to the target events (Addis et al., 2011; St Jacques et al., 2012). Functional neuroimaging studies investigate ng autobiographical memory under normal aging have observed increased activity in the hippocampus, parietal and occipital cortex during autobiographical memory recollection (Maguire and Frith, 2003; Donix et al., 2010), reduced activation in the prefrontal cortex and reduced coupling between the ventrolateral prefrontal cortex and hippocampus during search and elaboration phases of autobiographical memory retrieval (St Jacques et al., 2012), and decreased activity in the medial temporal lobes and precuneus during the construction of autobiographical events (Addis et al., 2011; St-Laurent et al., 2011).

In spite of some senescence-related deficits in autobiographical memory, normal older adults showed high levels of emotional stability and even superior emotional regulation (Kennedy et al., 2004). Socio-emotional Selectivity Theory (Carstensen et al., 2003) indicates that significant developmental changes occur in adults' regulation and processing of affect. As older adults begin to view their lifetime as limited, they show an increased motivation to attend to emotional stimuli and to evoke emotion regulation processes in favor of a maximization of positive compared to negative experiences, which has been referred to as an age-related “positivity effect” (Carstensen and Mikels, 2005). Previous behavioral studies suggest that this positivity effect that requires controlled processing and emotion regulation plays an important role in the lives of aged person (Mather and Knight, 2005; Knight et al., 2007).

There were numerous neuroimaging studies that investigated the neural mechanism underlying the emotional regulation in younger adults. It was found that the emotional regulatory processes were underlined by the involvement of control-appraisal system dynamics between the prefrontal cortex and the limbic regions. The prefrontal cortex includes the medial prefrontal cortex (PFC) (Ochsner et al., 2002; Delgado et al., 2008), lateral PFC (Ochsner et al., 2004; Delgado et al., 2008), and anterior cingulate cortex (ACC) (Ochsner and Gross, 2005; Delgado et al., 2008) and the limbic regions includes the amygdala (Ochsner et al., 2002; Delgado et al., 2008) and insula (Ochsner and Gross, 2005). In contrast, studies that explored the neural bases of emotion regulation in older adults are relatively fewer. Consistent with previous work in younger adults, it was reported that medial PFC and the amygdala were engaged in the emotional regulation of older adults (Urry et al., 2009). Urry et al. (2006) found that amygdala and ventromedial prefrontal cortex were inversely coupled during regulation of negative affect among older adults (Urry et al., 2006). A recent study (Winecoff et al., 2011) found that emotion regulation resulted in greater inverse connectivity between the left inferior frontal gyrus and amygdala for both younger and older adults and greater regulation-related activation in the left inferior frontal gyrus and the left superior temporal gyrus for younger vs. older adults. Moreover, functional neuroimaging studies investigating age-related differences in emotional processing found that older adults show an alteration in the recruitment of the amygdala and greater involvement of the frontal cortex, which was termed Fronto-amygdalar Age-related Differences in Emotion (FADE) by St Jacques et al. (2009a). Consistent with the FADE pattern, many studies have discovered that the age-related increase in PFC activity was always accompanied by down-regulated activity in the amygdala during the perception (Iidaka et al., 2002; St Jacques et al., 2010) and retrieval (Murty et al., 2009) of negative stimuli, which implyed that normal aging might be associated with a shift from automatic to more controlled emotional processing or emotion regulation via the additional recruitment of the PFC and decreased recruitment of the amygdala (Williams et al., 2006). A study that investigated the neural mechanism underlying the positivity effect demonstrated that ACC activation was related to the attentional positivity effect and emotional stability in successful aging (Brassen et al., 2011). Notably, several studies observed aged-related reversal in the valence of emotional images eliciting activity in the VMPFC (Leclerc and Kensinger, 2008, 2010) and emotional words eliciting activity in the amygdala and VMPFC/ACC (Leclerc and Kensinger, 2011). These results may suggest that age-related changes in the VMPFC/ACC contribute to older adults' positivity effect.

Recently, more and more studies have explored the impact of normal aging on emotional autobiographical memory and found that the positivity effect also existed in emotional autobiographical memory. Studies showed that older adults reported greater positive emotion in mutual reminiscing (Pasupathi and Carstensen, 2003; Comblain et al., 2005) and remembered past emotions as more positive and less negative (Kennedy et al., 2004) than the younger adults. Moreover, older adults recollected fewer negative memories in response to negative cues (Ros and Latorre, 2010), and more positive events to positive cues (Tomaszczyk and Fernandes, 2012) relative to their younger counterparts. These positivity effects observed in the older adults' emotional autobiographical memory could be attributed to superior emotion regulation processes in the elderly (Carstensen et al., 2003; Kennedy et al., 2004; Comblain et al., 2005).

Furthermore, several studies investigated the alteration of brain networks induced by normal aging. Numerous studies revealed that normal aging could result in disruptions of the DMN (default mode network) (Andrews-Hanna et al., 2007; Grady et al., 2012). Except the DMN, some other networks, such as reward network (Grady et al., 2012), motor network (Wu et al., 2007) and attention network (Andrews-Hanna et al., 2007), could also be affected by normal aging. Nowadays, a few studies investigated the age-related alterations in memory networks. St Jacques et al. (2009b) employed a subsequent memory paradigm of negative pictures and found that older adults showed decreased connectivity between amygdala and hippocampus, but increased connectivity between the amygdala and dorsolateral prefrontal cortices. Moreover, Sambataro et al. (2012) investigated age-related differences in brain networks during working memory and incidental episodic encoding memory and found that older adults, relative to younger adults, showed greater activity in prefrontal-parietal-occipital network and DMN (Sambataro et al., 2012). These studies demonstrated the modulation effects of normal aging on brain networks.

Although a number of studies investigated the neural bases of age-related alterations in autobiographical memory and emotional information processing independent of each other, to date, the effect of both emotionality and age on the neural mechanisms of emotional autobiographical memory has been relatively unexplored. More importantly, the neural mechanisms underlying the positivity effect observed in behavioral studies of emotional autobiographical memory remain unclear. To this end, the current study aims to investigate age- and valence-related alterations in both the behavior and neural correlates of autobiographical memories with different emotional valence. Because investigating both age and valence in one study can reveal the interaction effect between the two factors, the second purpose of this study is to further explore the neural basis of the positivity effect of emotional autobiographical memory. To address the above issues, we tested younger and older female adults using functional magnetic resonance imaging (fMRI) and an experimental paradigm similar to Viard et al. (2007).

Independent component analysis (ICA) (McKeown and Sejnowski, 1998) was applied to dissociate the brain networks related to each memory condition and to determine how those networks were affected by aging and emotional valence. Although the most widely used statistical approach to reveal brain region responses during cognitive tasks is the univariate general linear model (GLM), the GLM approach is unable to examine interactive and coordinated responses from multiple brain regions. Moreover, it has been demonstrated that cognitive processes depend on the integrated activity of spatially distributed brain regions, rather than the activity of any single brain region independently. Complementary to univariate approaches, multivariate statistical methods, such as ICA, consider brain voxels or regions of interest (ROI) as an integrated network and analyze them jointly. Within the context of fMRI, ICA is capable of identifying spatially distributed networks that are engaged in various elements of cognitive process without any prior knowledge, making it an increasingly attractive exploratory tool to study functional brain networks at rest or during a cognitive task.

Based on the age-related positivity effect in emotional autobiographical memory (Kennedy et al., 2004; Comblain et al., 2005), we would expect that older adults experienced greater positive feelings in autobiographical retrieving relative to their younger counterparts. We further hypothesized that the brain networks participating in the emotional autographical memories showed age- and valence-related alterations. Especially, the activity in the VMPFC/ACC of the task-related networks might show a saliently age-related reversal in emotional autobiographical retrieving and be associated with the positivity effect observed in behavior. Our rationale was that previous studies have consistently observed age-related reversal effect in the VMPFC/ACC region during emotional processing (Leclerc and Kensinger, 2008, 2010, 2011), and the VMPFC is a region implicated in valence-based processing (Leclerc and Kensinger, 2008, 2011) and emotion regulation (Ochsner et al., 2004; Cooney et al., 2007; Delgado et al., 2008). Finally, we hypothesize that the amygdala in the task-related networks might show an age-related change opposite to the VMPFC/ACC region during emotional autobiographical recollection, because the amygdala was reported to be inversely coupled with the MPFC during the emotion processing (Ochsner et al., 2002, 2004; Urry et al., 2006).

## Materials and methods

### Ethics statement

The study was approved by the Institutional Review Board of Beijing Normal University (BNU) Imaging Center for Brain Research, National Key Laboratory of Cognitive Neuroscience and Learning. The subjects gave written informed consent according to the guidelines of the MRI center at BNU.

### Subjects

Females differ from males at both the behavioral (Ros and Latorre, 2010) and neural level (Piefke and Fink, 2005) of autobiographical memory recall. To obtain a homogeneous group, we recruited only women in the current study. Twenty-seven right-hand dominant female subjects, including 13 younger adults (18–22 years of age) and 14 older adults (60–74 years of age) participated in the experiment. All subjects were native Chinese speakers with either normal or corrected-to-normal vision. The exclusion criteria included history of neurological or psychiatric disorders, head injury, and any stroke that may affect brain function. The mean years of education was 12.50 [standard deviation (*SD*) = 2.47] for the older adults and 13.61 (*SD* = 2.72) for the younger adults. The difference in education years between the two groups was not significant (*p* = 0.275). All of the older adults achieved scores that were higher than 27 on the Mini-Mental Status Examination (MMSE, Mean ± *SD* = 28.928 ± 0.916). The subjects gave written informed consent according to the guidelines of the MRI center at BNU. Two of the older adults were excluded from the analyses because they did not complete the task as instructed. Thus, the reported results are based on data from 13 younger (Mean age = 20.46, *SD* = 1.45; Mean education year = 13.61, *SD* = 2.72) and 12 older (Mean age = 65.42, *SD* = 4.56; Mean education year = 12.16, *SD* = 2.52) subjects.

### Task and experimental design

#### Pre-scan interview

Semi-structured autobiographical interviews with all subjects were carried out 1 month prior to fMRI scanning. Because explicit evidence indicates that different neural mechanisms underlie recent vs. remote memories (Viard et al., 2007), only recent memories from the past 5 years were taken into account in the current study. The mean duration of the autobiographical interview was 1 h. Subjects were asked to report 10 events that they could vividly remember from their recent past (the last 5 years before the interview): 5 with a positive content and 5 with a negative content. Subjects were required to provide the valence intensity of the events and the date when the events occurred. If they could not remember the specific date, they could provide an approximate date. The following instructions were given: “Please provide memories of a specific event that occurred within the last 5 years. The event you report must be one in which you were personally involved, and you must have a recollection of being personally involved in it. Do not pick events that you heard about from others. The events should occur at a specific time and place. Only events that lasted less than a day and did not frequently repeat over time can be reported.” During the experiment, the subjects spoke about the event without any interruption from the experimenter, who wrote down a description of the event and continued until it was evident that the subject had reached a natural ending point. Then, the experimenter checked the event to make sure that it contained sufficient details and asked the subject to provide additional information for events that were general in nature. For example, it would not be sufficient if the subject could not generate a specific memory and simply reported “playing table tennis in school.” The experimenter would provide some general cues such as “Could you report more details about this event?” or specific cues such as “Do you remember the weather when this event occurred?.” The remembered events were then used to create event-specific stimulus sentences, one sentence per autobiographical episode retrieved, that were presented during fMRI scanning in order to trigger the corresponding autobiographical memories. Thus, there were five sentences generated for each memory condition (positive or negative) for each subject.

#### Scanning session

**Figure 1:**
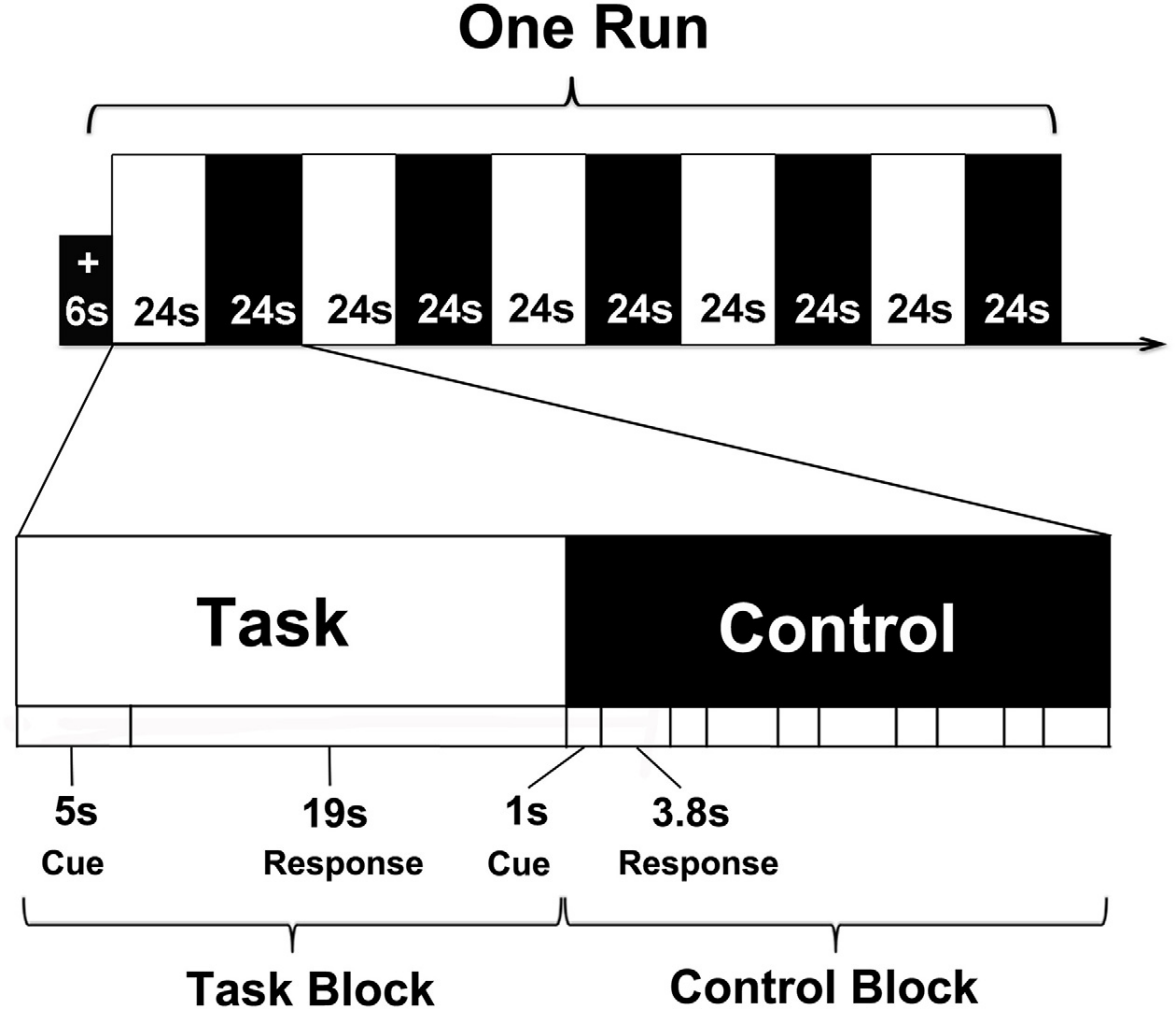
**fMRI experimental paradigm**.

Because the task-related components can be more reliably extracted from fMRI data of block design compared to the event-related design by ICA, the fMRI experiment in this study was designed in a blocked fashion that was similar to the paradigm used in a previous study (Viard et al., 2007) (Figure 1). Prior to the fMRI scan, each subject was required to participate in a pre-scan practice that consisted of one task block and one control block so that the subjects can understand the whole experimental procedure. The cue-sentences used in the task blocks of the pre-scan practice were constructed by the experimenters and unknown to the subjects. The control blocks were the same as the control blocks (described below) used in the actual scanning. During scanning, the cue-sentences, which were constructed from the subjects' prior interviews, were visually presented in white in the center of a black screen. An example of cue-sentence for a negative memory is “I cut my finger when I did sewing for the first time,” and for a positive memory, “Someone sent me four flower bouquets last Singles' Day.” All of the cue-sentences were presented in Chinese. The scanning session consisted of two functional runs with each run corresponding to one type of memory (i.e., positively or negatively toned memories). According to the previous study (Viard et al., 2007), each functional run consisted of 5 task blocks and 5 control blocks. Each block lasted for 24 s. At the beginning of each run, a central fixation cross was displayed on the screen for 6 s. The order of the five cue-sentences presented in each run was random. For each task block, one cue-sentence was presented for 5 s and followed by a black screen for 19 s. During the 5-s cue-sentence, the subjects were required to press a button as soon as they gained access to the event corresponding to the cue-sentence. In the following 19 s, the subjects were asked to recall and maintain the memory in mind as vividly as possible until the end of the block. Note that the order of the functional runs representing the two different memory types was counterbalanced across subjects.

In the control condition, the subjects were asked to examine a meaningless phrase composed of five Chinese characters and to react when a meaningless pseudo-character was present in the group (target group). The position of the pseudo-character in the meaningless phrase was random. Five meaningless phrases were presented during each control block and each phrase was presented for 1 s. In the following 3.8 s, the subjects were required to press a button if they detected the presence of a pseudo-character. Among the five meaningless phrases, only one phrase included a pseudo-character. The order of the target phrase was randomly intermixed across blocks (Figure 1). The control blocks were used to control for mental effects in the task blocks such as reading and motor processing.

#### Post-scan interview

Immediately following the scanning sessions, a post-scanning debriefing took place to verify each subject's engagement during scanning and to specify the nature of the events recalled during scanning. Using the same cue-sentences employed in the scanning sessions, the subjects were instructed to retrieve each of the 10 memories evoked in the scanner and asked to indicate whether they successfully recollected each event. In addition, all of the subjects were asked to rate their retrieved memories in terms of the intensity of emotional valence, the vividness of the visual imagery, the frequency of rehearsal (how frequently an event was recalled in daily life prior to scanning), and the personal significance using a 5-point scale (Appendix). These behavioral measures were selected for the following reasons: Firstly, we wanted to investigate valence-related changes when retrieving emotional autobiographical memories, and to do so, it is indispensable to estimate the subjective emotional valence experienced by the subjects. Secondly, visual imagery is one of the fundamentally essential cognitive processes of autobiographical retrieval, and vivid visual imagery increases the recall of specific details and the subjective sense of remembering (Dewhurst and Conway, 1994). Thirdly, several reports have demonstrated that the repetition of particular events affects autobiographical memories through the process of memory consolidation and results in the semantization of memories (Cermak, 1984). Finally, the personal significance of the event is thought to have an important role in re-experiencing by enabling mental time-travel of the self (Addis et al., 2004).

### Functional MRI data acquisition

Brain scans were performed at the MRI Center of BNU using a 3.0-T Siemens whole-body MRI scanner. A single-shot, T1^*^-weighted, gradient-echo EPI sequence was used for the functional imaging acquisition with the following parameters: 2000 ms repetition time, 30 ms echo time and 90° flip angle; 64 × 64 acquisition matrix; 200 × 200 mm field of view (FOV); and 4 mm slice thickness with a 0.8 mm inter-slice gap. Thirty-two axial slices parallel to the AC-PC line were obtained in an interleaved order covering the whole cerebrum and cerebellum.

### Data analysis

#### Behavioral data analysis

Two older adults were excluded from analysis because they failed to complete the task as instructed. Thus, 12 older subjects and 13 younger subjects were included in the following behavior and fMRI analyses. Using SPSS (version 16.0, http://www.spss.com), Two-Way repeated measures ANOVA with age as the between-subjects factor and emotional valence as the within-subject factor was conducted to assess potential age-related behavioral changes in the valence intensity, vividness, frequency of rehearsal, and personal significance of the post-scan interview and the valence intensity of the pre-scan interview.

#### Functional image analysis

The first three volumes of each subject's functional images that corresponded to the fixation stage were discarded to remove the instability of the scanner. SPM8 (http://www.fil.ion.ucl.ac.uk/spm/) was used for functional image preprocessing including realignment, normalization and smoothing. For each subject, the functional images were spatially realigned to correct for head motion and normalized to a standard EPI template (MNI brain). The functional data were then re-sampled into 3^*^3^*^3 mm^3^ voxels and smoothed using an isotropic Gaussian kernel of 8 mm full width at half maximum (FWHM). For each run of each group, the pre-processed data of all subjects were entered into GIFT (http://icatb.sourceforce.net) to perform group ICA, which includes 2-fold dimension reduction by principle component analysis (PCA), ICA decomposition, and back reconstruction. The optimal number of independent components, which were 33, 34, 31, and 30 corresponding to positive memories of the older adults, negative memories of the older adults, positive memories of the younger adults and negative memories of the younger adults, were selected to preserve at least 99% of the original variance on average. In the first PCA stage, the individual functional data of each run were reduced to the optimal number. After concatenation across subjects, the data were again reduced to the equal number of the first PCA stage through the second PCA stage. The data were then decomposed by ICA using the Infomax algorithm (Bell and Sejnowski, 1995). The ICA processing was repeated 50 times using ICASSO (http://research.ics.aalto.fi/ica/icasso/) to ensure the reliability of the component estimation. After ICA processing, a series of independent components together with their associated time courses for each run of each group were created. Back reconstruction was used to produce the individual time courses and spatial maps for every subject's functional data. We then used the correlation coefficient between the reference function and the mean time course of each independent component to determine the task-related independent components. The reference function was derived by convoluting the ON-OFF stimulus paradigm and the hemodynamic response function. Only those components with correlation coefficients above 0.43 (*p* < 0.000001) (Celone et al., 2006) were considered to be task-related and were included in further group analyses. The subsequent group analysis was conducted based on the random effects model in SPM8. The task-related components extracted in the former step were submitted to a full factorial model using age (between-subjects, older and younger) and emotional valence (within-subject, positive and negative) as the two main factors. For the baseline contrasts, the statistical threshold was corrected for multiple comparisons using the false discovery rate (FDR) at an overall (corrected) alpha level of 0.05 with a minimum cluster of 5 contiguous significant voxels. The contrasts of the main effect, interaction effect, and simple effect that were masked by the regions showing significant activation for either baseline contrast (*p* < 0.005, uncorrected) were corrected for multiple comparisons through FDR at an overall (corrected) alpha level of 0.05 with a minimum cluster of 5 contiguous significant voxels.

**Figure 2:**
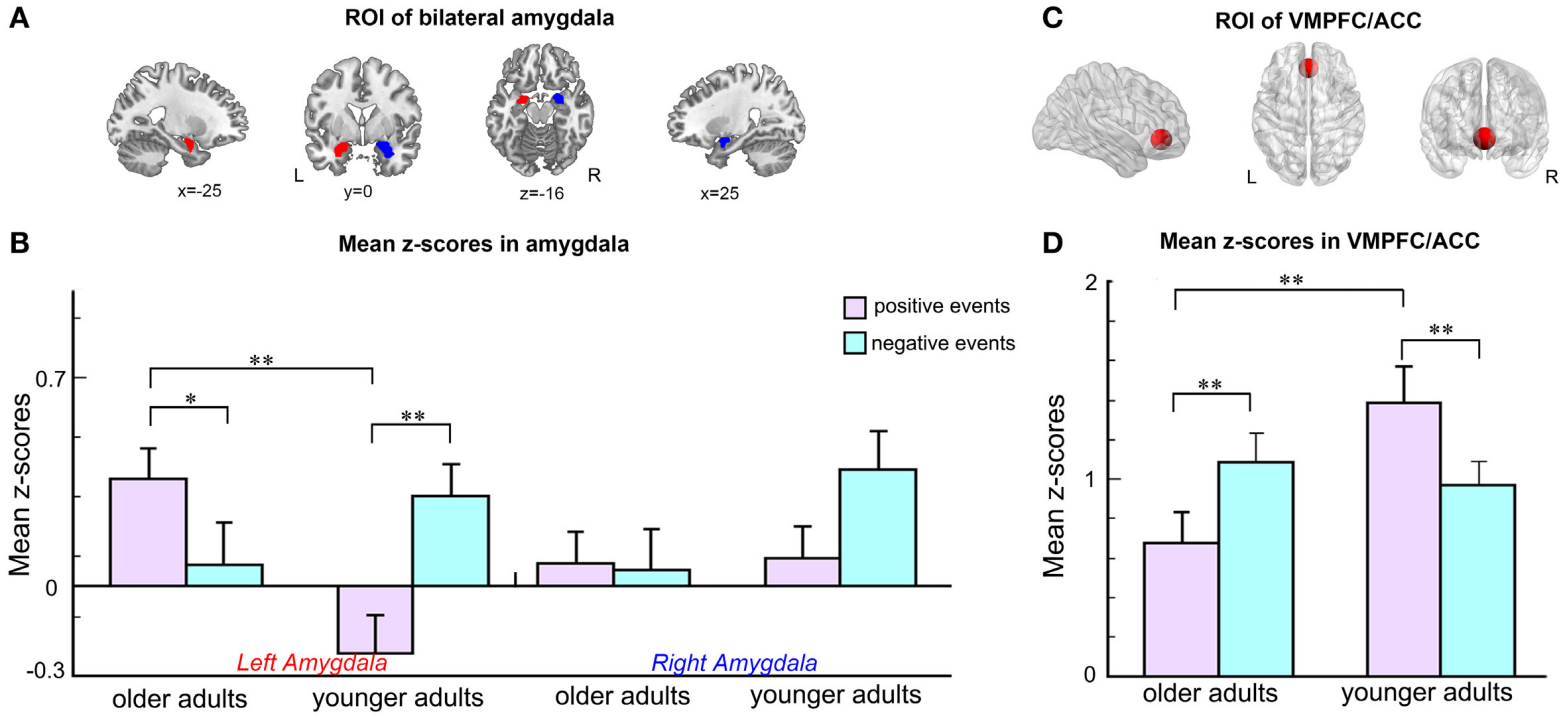
**Results of the ROI analyses for the region of the amygdala and the ventromedial prefrontal/anterior cingulate cortex. (A)** Coronal, axial and sagittal views showing the anatomically defined ROIs of the left (red) and right (blue) amygdala. **(B)** Average z-scores within the bilateral amygdala of the two groups during the positive and negative memory retrieval. **(C)** Coronal, axial and sagittal views showing the selected ROI of the VMPFC/ACC. **(D)** Average z-scores within the VMPFC/ACC of the two groups during the positive and negative memory retrieval. Error bars represent standard error values. ^**^p < 0.05; ^*^0.05 < *p* < 0.1.

Given the strong evidence that the amygdala and VMPFC/ACC play important roles during emotional perception and emotional episodic memory processing (Leclerc and Kensinger, 2008; St Jacques et al., 2009a), the amygdala and VMPFC/ACC were selected as ROIs for the assessment of age-related and valence-related differences during emotional autobiographical retrieval. Two ROIs of the left and right amygdala were created based on the automated anatomic labeling (AAL) brain template using the Wake Forest University (WFU) Pick-Atlas toolbox (http://www.fmri.wfubmc.edu/cms/software, version 3.0) (Figure 2A). The mean z-scores of these ROIs were extracted from each subject's task-related component. A repeated measures ANOVA with age (older vs. younger) as the between-subjects factor and emotional valence (positive vs. negative) as the within-subject factors was conducted on the the left and right amygdala, respectively, using SPSS 16.0. For the VMPFC/ACC, an ROI was defined as a sphere with a 12 mm radius centered on the coordinates reported in a previous study (*x* = 0, *y* = 39, *z* = −5 in Talairach space) (Leclerc and Kensinger, 2008) (Figure 2C). The mean z-score of the ROI was individually extracted from the task-related component of each subject. A repeated measures ANOVA with age (older vs. younger) as the between-subjects factor and emotional valence (positive vs. negative) as the within-subject factor was conducted on the VMPFC/ACC using SPSS 16.0.

A whole brain linear regression analysis was performed in SPM8 to reveal the brain regions that were correlated with the valence intensity of the retrieved events. For each group, the individual components related to positive event and those related to negative event retrieval were entered into SPM8 using the valence intensity as the regressor. This statistical analysis was corrected for multiple comparisons at the cluster level in the whole-brain statistics, yielding an overall corrected alpha rate of *p* < 0.05. The correction threshold was determined from a Monte Carlo simulation in AFNI and required a voxel-wise threshold of *p* < 0.001 within a minimum 3D cluster of 25 contiguous significant voxels.

## Results

### Behavior results

**Figure 3:**
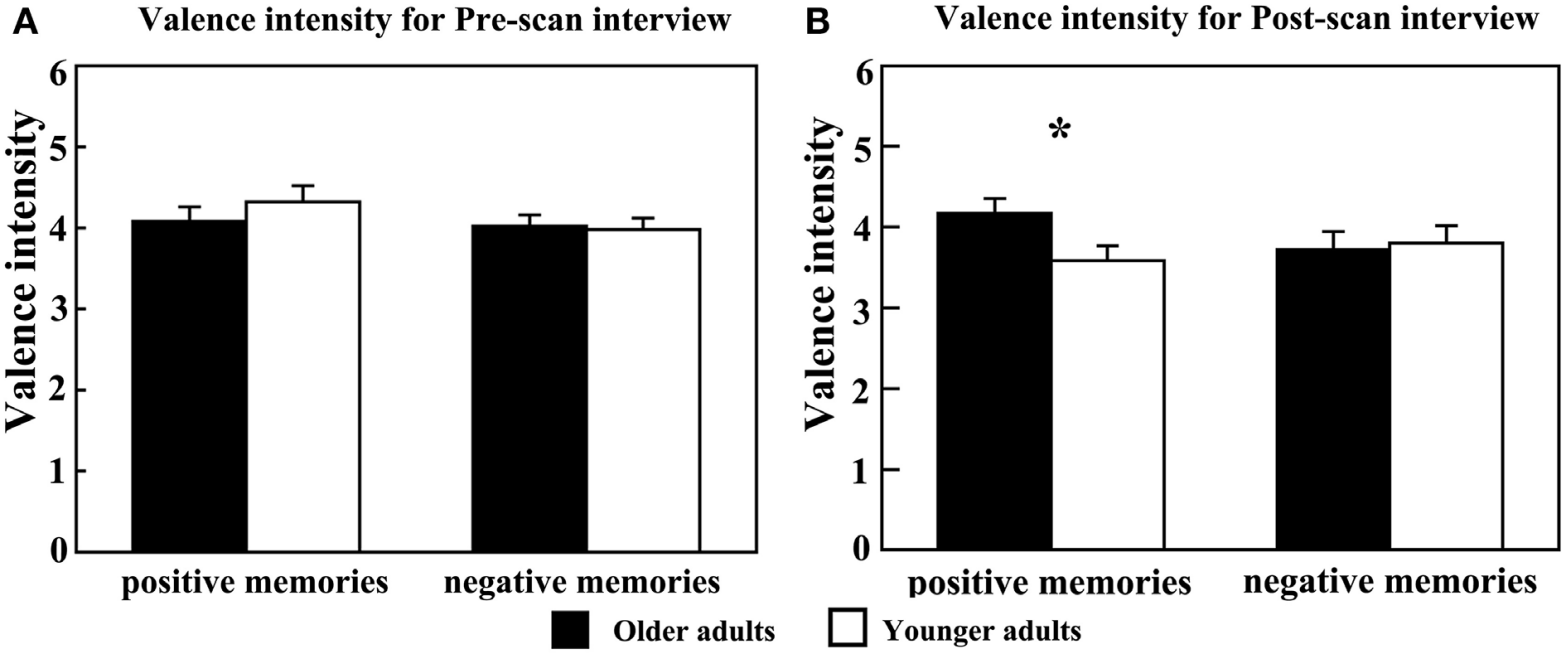
**Results of behavioral data in (A) pre-scan and (B) post-scan interviews in terms of emotional valence intensity.** ^*^p < 0.05. The error bars represent standard error values.

The results of ratings upon valence intensity in the pre-scan and post-scan interview are presented in Figure 3. For valence intensity of the pre-scan interview, analysis of variance (ANOVA) did not reveal significant main effect of age [*F*_(1, 23)_ = 0.249, *p* = 0.623], main effect of emotion [*F*_(1, 23)_ = 1.823, *p* = 0.190], and interaction effect [*F*_(1, 23)_ = 1.005, *p* = 0.326]. There was a marginally significant interaction between age and emotion [*F*_(1, 23)_ = 3.882, *p* = 0.061], whereas the main effects of age [*F*_(1, 23)_ = 1.119, *p* = 0.301] and emotion [*F*_(1, 23)_ = 0.483, *p* = 0.494] did not reach significance. Simple effect analysis indicated that positive memories of the older adults elicited significantly higher positive feelings than the younger adults [Mean ± *SE*: 4.167 ± 0.190 vs. 3.585 ± 0.182, *F*_(1, 23)_ = 4.890, *p* = 0.037]. As for negative memory retrieval, there was no significant difference between the two groups [*F*_(1, 23)_ = 0.070, *p* = 0.793]. Furthermore, older adults reported marginally significantly higher valence strength in positive vs. negative events [Mean ± *SE*: 4.167 ± 0.190 vs. 3.717 ± 0.227, *F*_(1, 11)_ = 3.415, *p* = 0.078]. With regard to vividness, the ANOVA test disclosed marginally significant main effect of age [*F*_(1, 23)_ = 3.547, *p* = 0.072] and emotion [*F*_(1, 23)_ = 3.261, *p* = 0.084], which indicated that the older adults reported more vividly than the younger ones [Mean ± *SE*: 4.192 ± 0.185 vs. 3.708 ± 0.178] and that positive memories are more vivid than negative ones [Mean ± *SE*: 4.116 ± 0.100 vs. 3.783 ± 0.200]. The interaction between age and emotion was not significant [*F*_(1, 23)_ = 0.405, *p* = 0.531]. As for the respective frequency of rehearsal and personal significance, there was no significant age effect [*F*_(1, 23)_ = 0.581, *p* = 0.454; *F*_(1, 23)_ = 0.249, *p* = 0.623], emotion effect [*F*_(1, 23)_ = 1.914, *p* = 0.180; *F*_(1, 23)_ = 1.823, *p* = 0.190], and interaction effect between age and emotion [*F*_(1, 23)_ = 0.256, *p* = 0.618; *F*_(1, 23)_ = 1.005, *p* = 0.326].

### fMRI results

#### Brain networks associated with negative/positive memory retrieval

**Figure 4:**
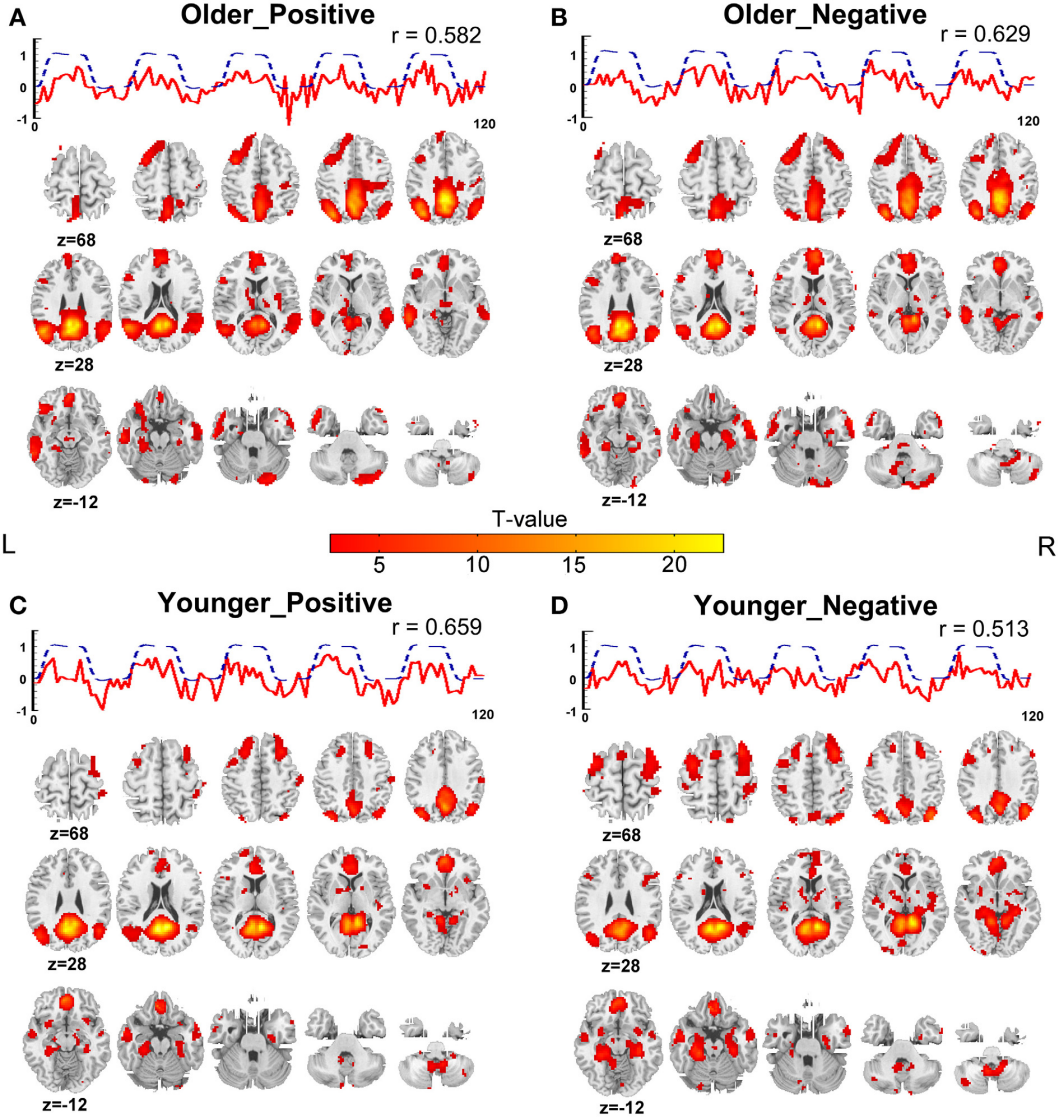
**Spatial activation maps of the task-related components and associated time courses. (A)** Positive memory retrieval of the older adults. **(B)** Negative memory retrieval of the older adults. **(C)** Positive memory retrieval of the younger adults. **(D)** Negative memory retrieval of the younger adults. The statistical threshold was set at *p* < 0.05, corrected for multiple comparisons through FDR with a minimum cluster of 5 contiguous significant voxels. The solid line indicates the time courses of task-related components, and the dotted line indicates the reference function. *r* represents the correlation coefficient between the time course of the task-related component and the reference function.

For each group, one component related to the retrieval of positive autobiographical memories and one component related to the retrieval of negative autobiographical memories were extracted using the ICA method. The spatial mappings and the corresponding time courses of the task-related networks for the two groups are shown in Figure 4. A full account of the regions participating in the brain networks of positive or negative autobiographical memory retrieval is given in Table 1 for the older adults and Table 2 for the younger adults. It can be seen that all of the task-related networks showed similar activation patterns. Robust activation in the medial and lateral frontal cortex, ACC, hippocampus/parahippocampus, middle and superior temporal cortex, lateral parietal cortex, precuneus, retrosplenial/posterior cingulate cortex and cerebellum were observed in the task-related networks of the two groups.

**Table 1:** **Activated brain regions of the components associated with the positive and negative autobiographical memory tasks for older adults**.

**Regions**	***BA***	**Positive memory**				**Negative memory**			
		***x***	***y***	***z***	***T*_max_**	***x***	***y***	***z***	***T*_max_**
Left medial frontal gyrus	10	−3	50	16	7.34	−3	47	−8	7.87
Right medial frontal gyrus	9/10	6	50	19	5.70	9	47	10	6.01
Left inferior frontal gyrus	47	−33	29	−17	4.19	−30	30	−11	3.74
Left middle frontal gyrus	6/8	−39	11	55	8.64	−24	29	46	5.14
Right middle frontal gyrus	6/8					30	23	52	5.50
Left anterior cingulate	24/32	−3	38	−8	4.12	−3	44	1	7.01
Right anterior cingulate	24/32					3	41	1	6.79
Left hippocampus		−27	−7	−17	3.30	−27	−19	−17	4.85
Right hippocampus						18	−13	−17	4.17
Left parahippocampus	35/36	−27	−31	−17	4.87	−27	−31	−17	4.73
Right parahippocampus	35/36	30	−28	−17	3.64	21	−19	−17	5.18
Left amygdala		−30	−4	−20	3.36				
Left thalamus		−9	−13	13	4.13	−9	−10	10	3.62
Right thalamus		6	−16	−2	3.21	12	−13	7	4.11
Right insula	13	33	−7	13	4.80	45	−10	−2	3.31
Left middle temporal gyrus	21	−60	−19	−23	8.36	−63	−37	−5	5.73
Right middle temporal gyrus	21	54	2	−26	4.16	60	−7	−20	5.58
Left temporoparietal junction	39	−48	−61	28	13.93	−45	−61	25	9.02
Right temporoparietal junction	39	48	−61	28	6.67	54	−61	26	6.17
Left superior parietal lobule	7	−33	−70	52	4.79	−39	−73	49	6.62
Left inferior parietal lobule	39/40	−36	−73	40	13.94	−45	−64	34	8.46
Right inferior parietal lobule	39/40	45	−67	37	7.11	51	−67	31	6.83
Left retrosplenial/posterior cingulate	23/29/30/31	−6	−55	16	13.44	−3	−52	16	15.15
Right retrosplenial/posterior cingulate	23/29/30/31	9	−55	16	13.82	6	−52	13	17.36
Left precuneus	7/31	−3	−49	34	20.61	−6	−52	34	17.00
Right precuneus	7/31	6	−64	40	8.85	3	−61	34	14.06
Left cerebellum		−6	−55	−50	4.87	−18	−64	−35	4.20
Right cerebellum		27	−82	−29	6.11	9	−49	−47	5.74

The displayed local maxima were corrected with p < 0.05, FDR; MNI coordinates; Abbreviations: BA, Brodmann's area.

**Table 2:** **Activated brain regions of the components associated with the positive and negative autobiographical memory tasks for younger adults**.

**Regions**	***BA***	**Positive memory**				**Negative memory**			
		***x***	***y***	***z***	***T*_max_**	***x***	***y***	***z***	***T*_max_**
Left medial frontal gyrus	10	−6	47	−11	11.30	−3	53	−5	7.06
Right medial frontal gyrus	10	3	47	−8	8.19	6	50	−5	4.72
Left middle frontal gyrus	6/8	−24	20	49	5.88	−27	17	52	4.99
Right middle frontal gyrus	6/8	27	20	49	6.34	27	20	46	7.44
Left anterior cingulate	24/32	−6	38	1	6.66	−6	38	4	4.08
Right anterior cingulate	24/32	3	35	7	5.54	6	38	7	3.44
Left hippocampus		−24	−19	−17	4.11	−30	−19	−20	5.95
Right hippocampus		30	−13	−20	3.40	21	−13	−20	3.90
Left parahippocampus	35/36	−30	−31	−17	5.81	−24	−31	−20	8.59
Right parahippocampus	35/36	30	−19	−26	4.97	24	−16	−26	5.24
Left amygdala						−30	−1	−17	4.83
Right amygdala						30	2	−17	3.53
Left thalamus		−18	−4	7	3.10	−12	−10	1	4.29
Right thalamus						6	−10	10	3.67
Left insula	13					−39	−22	7	3.56
Right insula	13	42	−22	7	3.77	42	−4	4	4.25
Left middle temporal gyrus	21	−54	−4	−20	5.51	−51	−7	−17	5.93
Right middle temporal gyrus	21	54	−10	−20	3.85	57	−4	−23	4.86
Left temporoparietal junction	39	−45	−61	25	5.72	−45	−70	28	5.62
Right temporoparietal junction	39	45	−61	28	7.15	48	−64	25	6.21
Left superior parietal lobule	7	−30	−76	46	4.28	−27	−76	52	3.55
Right superior parietal lobule	7	33	−76	49	5.65	30	−73	49	5.84
Left inferior parietal lobule	39/40	−42	−76	37	8.27	−36	−79	40	8.64
Right inferior parietal lobule	39/40	42	−70	40	7.00	42	−70	40	10.35
Left retrosplenial/posterior cingulate	23/29/30/31	−6	−58	13	13.72	−12	−55	10	15.91
Right retrosplenial/posterior cingulate	23/29/30/31	6	−58	10	14.51	9	−52	10	19.12
Left precuneus	7/31	−6	−58	22	20.56	−6	−61	25	14.74
Right precuneus	7/31	12	−61	28	9.90	3	−67	34	11.82
Left cerebellum		−6	−52	−44	5.11	−6	−52	−41	6.43
Right cerebellum		9	−49	−47	8.03	15	−43	−50	6.49

The displayed local maxima were corrected with p < 0.05, FDR; MNI coordinates; Abbreviations: BA, Brodmann's area.

#### Age- and valence-related alterations of the task-related networks

**Figure 5:**
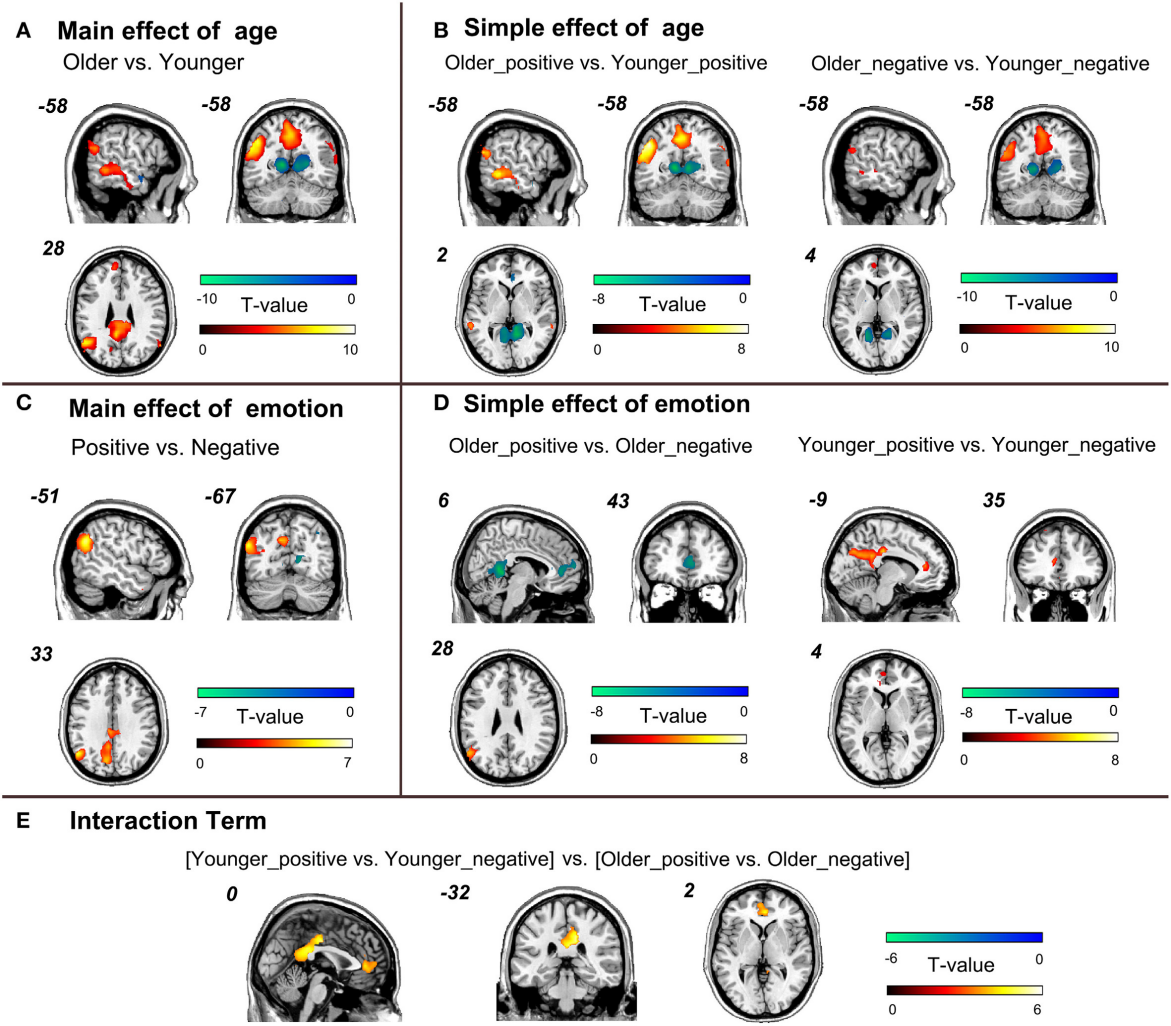
**Brain regions showing significant main, simple, and interaction effect of age and emotion. (A)** Brain regions showing significantly main effect of age. **(B)** Brain regions showing significantly simple effect of age. **(C)** Brain regions showing significantly interaction between age and emotion. **(D)** Brain regions showing significantly simple effect of emotion. **(E)** Brain regions showing significant interaction effect of age and emotion. The statistical threshold was set at *p* < 0.05, corrected for multiple comparisons through FDR with a minimum cluster of 5 contiguous significant voxels.

Compared to the younger adults, the older adults showed greater activities in the left lateral parietal lobule, bilateral precuneus and left lateral temporal cortex of the brain network engaged in autobiographical memory recollection, regardless of the emotional valence (Figure 5A). By contrast, the younger adults exhibited higher recruitment of the right frontal cortex and bilateral retrosplenial/posterior cingulate within the task-related network relative to the older adults (Figure 5A). The coordinates and statistics of significant inter-group differences for positive/negative memory retrieval are presented in Table 3. The simple effect analyses indicated that the brain activation of the older adults was significantly greater in the bilateral precuneus, left lateral parietal cortex, and lateral temporal cortex within the task-related network than that of the younger adults for positive memory retrieval. In contrast, the younger group manifested significantly stronger activation in the VMPFC, bilateral retrosplenial cortex/posterior cingulate cortex, right anterior cingulate and right dorsolateral prefrontal cortex during the retrieval of positive memories. For negative memory retrieval, the brain regions showing inter-group differences were similar to those for positive memory retrieval. Stronger activities in the VMPFC, precuneus, lateral temporal cortex and lateral parietal cortex were observed for older vs. younger adults. In contrast to the older group, the younger group produced greater activity in the retrosplenial cortex/posterior cingulate cortex and middle frontal cortex (Figure 5B, Table 3).

**Table 3:** **Relative increases in brain activity between older and younger adults during positive and negative autobiographical memory retrieval**.

**Regions**	***BA***	**Main effect of age: Older > Younger**			
		***x***	***y***	***z***	***T*_max_**
Left inferior parietal lobule	39/40	−54	−58	31	7.92
Left precuneus	7/31	−6	−49	34	8.96
Right precuneus	7/31	3	−49	49	8.01
Right middle cingulate	31	3	−34	43	8.89
Left middle temporal gyrus	21/22	−63	−43	−11	6.47
LEFT medial frontal gyrus	10	6	59	22	5.20
		**Main effect of age: Younger > Older**			
Right superior frontal gyrus	8	24	17	49	4.41
Left orbital gyrus	11	−3	38	−23	5.51
Left retrosplenial/posterior cingulate	29/30	−12	−61	7	7.52
Right retrosplenial/posterior cingulate	29/30	12	−52	4	6.62
Left cerebellum		−12	−52	−41	4.29
		**Older-Positive > Younger-Positive**			
Left inferior parietal lobule	39/40	−36	−67	40	5.25
Left precuneus	7/31	−3	−49	37	7.52
Right precuneus	7/31	6	−52	46	6.49
Left middle temporal gyrus	21	−60	−46	−5	6.48
Right middle temporal gyrus	22	66	−40	−2	3.98
		**Older-Negative > Younger-Negative**			
Left inferior parietal lobule	39/40	−45	−58	40	5.74
Left precuneus	7/31	−6	−52	34	7.14
Right precuneus	7/31	6	−52	37	4.70
Left middle temporal gyrus	21	−66	−43	−8	4.66
Right cingulate gyrus	24	3	−22	40	9.44
Left medial frontal gyrus	10/32	−3	53	13	4.97
		**Younger-Positive > Older-Positive**			
Right middle frontal gyrus	6/8	27	20	52	4.35
Left orbital gyrus	11	−6	41	−20	6.88
Right anterior cingulate	24/32	6	32	1	3.55
Left retrosplenial/posterior cingulate	29/30	−12	−61	7	6.17
Right retrosplenial/posterior cingulate	29/30	9	−52	7	6.70
		**Younger-Negative > Older-Negative**			
Right middle frontal gyrus	6/8	30	14	61	3.63
Left orbital gyrus	11	−6	41	−20	6.61
Left retrosplenial/posterior cingulate	29/30	−12	−58	7	6.43
Right retrosplenial/posterior cingulate	29/30	18	−52	4	5.49

The displayed local maxima were corrected with p < 0.05, FDR; MNI coordinates; Abbreviations: BA, Brodmann's area.

Regions showing main effect of emotion were located in the left precuneus and left temporoparietal junction, medial frontal gyrus and parahippocampus (Figure 5C, Table 4). For positive vs. negative memories, the activity within the VMPFC and ACC was stronger for the younger adults and weaker for the older adults. In addition, younger adults' activities within posterior cingulate cortex and precuneus were more intensive in positive vs. negative condition (Table 4 and Figure 5D).

**Table 4:** **Relative increases in brain activity between positive and negative autobiographical memory retrieval in older and younger adults**.

**Regions**	***BA***	**Interaction term [(YP > YN) > (OP > ON)]**			
		***x***	***y***	***z***	***T*_max_**
Right anterior cingulate	24/32	3	41	1	4.76
Left medial frontal gyrus	10/32	−3	53	4	4.15
Right middle cingulate	24/31	3	−19	37	5.92
Right posterior cingulate	31	6	−37	25	5.42
		**Main effect of emotion: Positive > Negative**			
Left precuneus	7	−9	−70	34	4.97
Left temporoparietal junction	39	−54	−64	28	6.66
Left middle cingulate	31	−3	−25	34	4.51
		**Main effect of emotion: Negative > Positive**			
Right medial frontal gyrus	10	6	62	13	5.00
Right parahippocampus	30	9	−43	1	5.40
		**Older-Negative > Older-Positive**			
Right anterior cingulate	32	3	41	4	4.53
Right medial frontal gyrus	10	9	62	10	4.37
Right posterior cingulate	29	6	−49	7	5.64
		**Older-Positive > Older-Negative**			
Left temporoparietal junction	39	−54	−58	31	5.31
		**Younger-Positive > Younger-Negative**			
Left medial frontal gyrus	10	−3	53	4	3.04
Left anterior cingulate	24/32	−6	38	16	4.68
Right middle cingulate	24	3	−22	37	6.39
Left precuneus	7	−9	−70	34	4.89
Left temporoparietal junction	39	−54	−64	28	4.20
		**Younger-Negative > Younger-Positive**			
No significant regions					

The displayed local maxima were corrected with p < 0.05, FDR; MNI coordinates; Abbreviations: BA, Brodmann's area; YP, Younger-Positive; YN, Younger-Negative; OP, Older-Positive; ON, Older-Negative.

An analysis of the interaction effect revealed that the younger adults displayed significantly greater activities in the VMPFC/ACC, middle and posterior cingulate region compared to the older adults for positive vs. negative memories (Table 4 and Figure 5E).

#### Results of ROI analysis

For the left amygdala, an ANOVA revealed a significant interaction of age and valence [*F*_(1, 23)_ = 13.575, *p* = 0.001]. No significant main effects of valence [*F*_(1, 23)_ = 1.182, *p* = 0.288] and age [*F*_(1, 23)_ = 1.743, *p* = 0.200] were found. The older adults showed marginally significantly greater activity in the left amygdala when comparing the positive events to the negative events [Mean ± *SE*: 0.360 ± 0.121 vs. 0.070 ± 0.130, *F*_(1, 23)_ = 3.243, *p* = 0.085]. In contrast, the left amygdala of the younger adults showed significantly stronger responses to the negative events than the positive events [Mean ± *SE*: 0.304 ± 0.124 vs. −0.227 ± 0.117, *F*_(1, 23)_ = 11.859, *p* = 0.002]. During the positive memory retrieval, older adults' left amygdala produced significantly greater activity than younger adults' left amygdala [Mean ± *SE*: 0.360 ± 0.121 vs. −0.227 ± 0.117, *F*_(1, 23)_ = 12.161, *p* = 0.002]. For the right amygdala, an ANOVA did not revealed significant main effect of valence [*F*_(1, 23)_ = 1.221, *p* = 0.281], main effect of age [*F*_(1, 23)_ = 2.093, *p* = 0.161], and interaction effect of age and valence [*F*_(1, 23)_ = 1.627, *p* = 0.215] (Figure 2B).

The analysis of the VMPFC/ACC showed that there were no significant main effects of valence [*F*_(1, 23)_ = 0.001, *P* = 0.978] and age [*F*_(1, 23)_ = 2.641, *p* = 0.118]. The interaction between these two factors was significant [*F*_(1, 23)_ = 13.020, *p* = 0.001]. Simple effect tests revealed that the negative events induced stronger activation in the older adults [Mean ± *SE*: 1.082 ± 0.138 vs. 0.670 ± 0.176, *F*_(1, 11)_ = 6.355, *p* = 0.019] and significantly weaker activation in the younger adults [Mean ± *SE*: 0.979 ± 0.132 vs. 1.385 ± 0.169, *F*_(1, 12)_ = 6.679, *p* = 0.017] than the positive events (Figure 2D). Furthermore, the younger adults' activity within this ROI was significantly higher than their older counterparts [*F*_(1, 23)_ = 8.531, *p* = 0.008] during the positive condition.

#### Results of regression analysis

**Figure 6:**
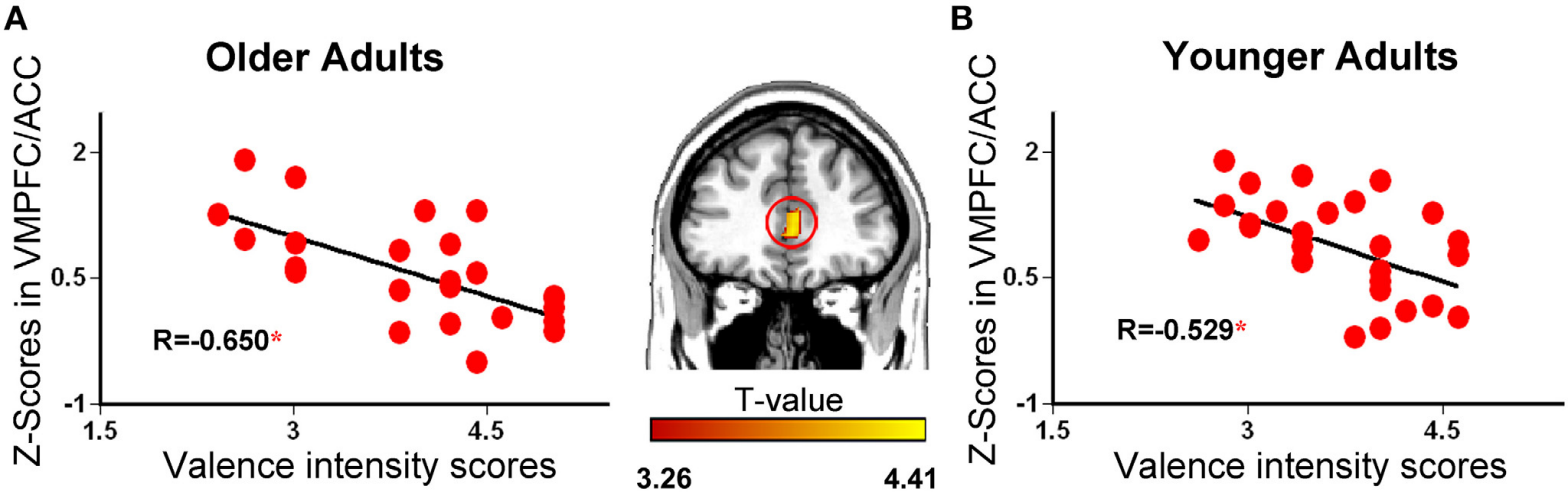
**Mean responses in the ventromedial prefrontal cortex (VMPFC) correlated significantly with the valence intensity of the retrieved autobiographical events in the older **(A)** and younger **(B)** groups for positive and negative memories, respectively.** The VMPFC region outlined in red (middle panel) was obtained by regression analysis using emotional valence intensity scores as the regressor. The value of *r* represents correlation coefficient with *p* < 0.01 (^*^).

A regression analysis revealed that only one cluster in the VMPFC/ACC (Figure 6; region marked by the red circle; MNI coordinate: 3, 38, 4) showed a significant negative correlation with valence intensity. We performed an additional correlation analysis to examine the relationship between VMPFC/ACC activation and valence intensity. For both groups, the activity in the VMPFC/ACC decreased linearly as the mean intensity of the events increased (Figure 6).

## Discussion

The current study provides evidence for differences at both the behavioral and brain network level between older and younger adults during an autobiographical memory task with positive and negative events. Older adults, compared to their younger counterparts, reported relatively higher positive feelings when retrieving emotional autobiographical events. Moreover, our functional imaging data revealed that the networks related to different emotionally toned memories showed opposite activity patterns of the VMPFC/ACC and amygdala for older vs. younger adults. Across all of the subjects and memory conditions, a regression analysis revealed that the degree of activity in the VMPFC/ACC correlated negatively with the valence intensity scores of the retrieved events. Our results suggest that older adults' “positivity effect” in emotional autobiographical retrieval may stem from changes in emotional processing implemented by the VMPFC/ACC and amygdala.

### Behavioral results

In line with recent behavioral studies (Kennedy et al., 2004; Comblain et al., 2005; Ros and Latorre, 2010), the post-scan interview revealed an intriguing bias toward positive emotion during the retrieval of autobiographical memories in older adults. Compared to the younger adults, the older adults rated emotional valence to be more positive in positively toned recollection. This “positivity bias” in the process of autobiographical event retrieval among the older adults is consistent with a wide range of aging studies (Mather and Carstensen, 2005) that observed a tendency for older adults to focus more on positive information. This effect was also found during autobiographical memory retrieval (Ros and Latorre, 2010). Our results further demonstrated that the bias toward positive feelings in autobiographical memory retrieval occurred with normal aging. It should be noted that valence intensity rated by the older group did not differ from the younger group for both the positive and negative events in the pre-scan interview. Our results suggest that the events recalled by both groups were not different from each other from the time of pre-scan interview. During the scanning, the participants were required to recall the events elaborately and maintain the memory in mind as vividly as possible. Under the influence of the prior scanning, the participants might process the events with more elaboration during the post-scan interview than the pre-scan interview. The result that the positivity effect took place at the post-scan interview rather than at the pre-scan interview might indicate that positivity effect occur consistently when adults process information in a sustained and elaborative way (Kensinger, 2008; Leclerc and Kensinger, 2010). Therefore, our behavioral results suggest that the age-related reversal in the VMPFC/ACC and amygdala were not due to the unequal valence of the events recalled by the two groups but rather to genuine distinctions in the neural substrates associated with the retrieval of emotional autobiographical memory.

In addition, the subjective measures of episodicity that was based on the subjects' ratings of the quality of visual mental imagery showed that autobiographical memories were recalled more vividly by the older adults relative to the younger adults. This result differs from previous studies reporting a decrease in reminiscent quality with age (Piolino et al., 2002). However, our study is not the first to report this surprising result. Other behavioral studies also showed that older adults reported a higher rating of vividness than younger adults (Rubin and Berntsen, 2009; Janssen et al., 2011). Moreover, it was recently reported that older vs. younger adults expressed higher scores of vividness (Donix et al., 2010) in a neuroimaging study on autobiographical memory. Higher scores of the quality of mental visual imagery in older adults may represent a trend for this age group to give higher ratings (Rubin and Berntsen, 2009) or a recalibration of the scales with age (Janssen et al., 2011). On the other hand, the disparity between our result and others might be attributed to the fact that our retrieval quality data and those of Donix's study (Donix et al., 2010) were collected during the post-scan debriefing rather than online.

### fMRI results

One task-related independent component was detected by the ICA method for each memory type (positively and negatively toned) in older and younger adults. As shown in Figure 4, the mean time courses of the four components across subjects were all highly correlated with the corresponding paradigm of the experimental tasks, implying that all of these networks were involved in the processing of autobiographical memory recollection. The spatial activation patterns of the four networks are largely overlapping (Figure 4). The commonly activated brain areas include the lateral and medial prefrontal cortex extending into the ACC, the lateral and medial temporal regions, the temporoparietal junction, the retrosplenial/posterior cingulate cortex with the adjacent medial parietal cortex, and cerebellum. All of these regions were reported to be engaged in autobiographical memory retrieval in previous studies (Svoboda et al., 2006; Cabeza and St Jacques, 2007). These common areas potentially reflect that the four brain networks participated in common cognitive processes of positive/negative autobiographical memory retrieval. For instance, the lateral and ventromedial prefrontal cortex may be engaged in memory search and monitoring processes, respectively, (Cabeza and St Jacques, 2007); the medial prefrontal cortex may participate in self-referential processes (Svoboda et al., 2006); and the posterior cingulate and medial temporal region may be responsible for the visuospatial processing and recollection (Svoboda et al., 2006). Moreover, it should be noted that the brain network engaged in the emotional autobiographical memory covered the core brain regions of the DMN that included retrosplenial/posterior cingulate, lateral parietal cortex, medial prefrontal cortex, lateral temporal cortex and hippocampal/parahippocampal regions. Generally, DMN can be extracted independently from task fMRI data by ICA and the time course of DMN is negatively correlated with the task paradigm for most cognitive tasks (Calhoun et al., 2008). However, the independent DMN was not dissociated from our data. Previous studies that investigated the relationship between the DMN and the activation pattern of autobiographical memory, prospection and theory of mind have demonstrated that the DMN supports common aspects of cognitive behaviors involved in simulation an internalized experience (Spreng and Grady, 2010). Thus, the regions within the DMN were observed to be activated by autobiographical memory task in this study. Moreover, except the brain regions in the DMN, the network participating in the emotional autobiographical memories included some other regions, such as amygdala and cerebellum (Svoboda et al., 2006), which were important to the emotional autobiographical memory.

It has been demonstrated that normal aging can result in reduced activity of the DMN (Damoiseaux et al., 2008; Sambataro et al., 2010). Many previous studies found that the retrosplenial/posterior cingulate cortex within the DMN showed decreased activity during normal aging (Koch et al., 2010; Sambataro et al., 2010). In this study, the older adults also exhibited weaker activation in the retrosplenial/posterior cingulate cortex within the task-related networks for both the positive and negative memories in contrast to the younger adults. The retrosplenial/posterior cingulate cortex is recognized as a core region of the autobiographical network (Svoboda et al., 2006) and is predominantly involved in supporting internally directed thought, especially with self-related processing (Leech and Sharp, 2014). When recalling autobiographical memories, the self-related events need to be retrieved. Since it was found that older adults showed deficits to “travel back in time” to relive their personal events (Piolino et al., 2006; St Jacques et al., 2012), age-related decrease in the retrosplenial/posterior cingulate cortex was observed in the study. Moreover, the activities of some brain regions within the task-related networks showed age-related increase. The inter-group comparison revealed that the older adults recruited the bilateral precuneus more in both positive and negative memories in contrast to the younger group. The precuneus was reported to be correlated with ratings of vividness (Gilboa et al., 2004) and play a key role in visual imagery processing in episodic memory recall (Fletcher et al., 1995; Cabeza and St Jacques, 2007). As our behavioral results showed that older adults recalled autobiographical memories more vivid than the younger adults, it is reasonable to observe greater activity in the precuneus of the older group than the younger group. Furthermore, there was an age-related increase in the left inferior parietal lobe within the task-related networks. The attention to memory (AtoM) hypothesis (Ciaramelli et al., 2008) suggests that the superior parietal cortex mediates the allocation of attentional resources to memory in a top-down way, and the inferior parietal cortex is associated with the mediation of the attention capture by recovered memory contents in a bottom-up way. Because older adults showed declines in their bottom-up attentional process (Madden, 2007), they might need greater involvement of the parietal cortex to offset the deficit of their bottom-up attentional processing compared to the younger adults.

The ROI analysis showed significantly greater activity in the VMPFC/ACC region of older adults but weaker activity in younger adults for negative vs. positive events retrieval. That is, the activity of the VMPFC/ACC exhibited an age-related reversal in valence-based autobiographical memories. Regression analyses revealed that the activity in the VMPFC/ACC negatively correlated with the valence intensity scores across the entire sample, which indicated that activity in this region was related to adults' valence intensity. As a result of the negative correlation between the activity of this region and the valence intensity, it was sound that the older adults ascribed higher emotional valence to positive retrieval than the younger adults. The finding of age-related reversal within the VMPFC/ACC region parallels previous findings (Leclerc and Kensinger, 2011). Due to the negative correlation between the activity of VMPFC/ACC and subject-rated valence, age-related valence-based reversal within the VMPFC/ACC region might be associated with older adults' positivity effect observed in our behavioral data. Previous studies demonstrated a link between older adults' positivity effect and controlled emotion processing or emotion regulation (Carstensen and Mikels, 2005; Leclerc and Kensinger, 2008; Kensinger and Leclerc, 2009). Older adults tend to process negative memories in a more positive way than younger adults do (Comblain et al., 2005), and they seem to devote a greater proportion of cognitive resources to emotional regulation (Kensinger and Leclerc, 2009), thereby allowing them to succeed at their regulation attempts (Urry et al., 2006; St Jacques et al., 2010). When older adults' cognitive control resources were limited, the positivity effect could not be observed in the older adults (Mather and Knight, 2005). Because the VMPFC/ACC is often associated with the regulation of both negative (Delgado et al., 2008; Kross et al., 2009) and positive (Beauregard et al., 2001; Hamilton et al., 2011) emotions, the current results suggest that adults' positivity effect may arise from age-related changes in controlled emotional processing or regulation implemented by the VMPFC/ACC.

Moreover, previous studies have revealed that the VMPFC plays a role in monitoring information retrieved from autobiographical memory (Cabeza and St Jacques, 2007). It was pointed out that older adults showed preserved memory for positive information, and poorer memory and fewer details for negative information compared to the younger adults (Mickley and Kensinger, 2009; Addis et al., 2010). In contrast to the positive autobiographical memory, older adults might need more efforts to retrieve negative memories. Thus, older adults possibly require more resources to monitor negative events than positive events, which may result in stronger activities in VMPFC/ACC of older adults during the negative retrieval.

It should be noted that the VMPFC was also associated with the self-referential processing (Northoff and Bermpohl, 2004). In contrast to processing information in reference to others, the activity in the VMPFC is greater when processing information in a self-relevant manner for both older and younger adults (Gutchess et al., 2007; Ruby et al., 2009). Because older adults are more likely to process positive information in reference to themselves compared to negative information (Kensinger and Leclerc, 2009), they should show greater activity in the VMPFC/ACC region during the processing of positive compared to negative information. Therefore, the weaker activity in the VMPFC/ACC of the older adults for the positive vs. negative retrieval in the present study did not support that age-related reversal in the VMPFC/ACC was connected with the self-referential process.

We also noted that two previous studies reported an age-related reversal in the valence-associated VMPFC/ACC activation that is contrary to our results (Leclerc and Kensinger, 2008, 2010). They found that in comparison with positive images, negative images activated the VMPFC/ACC more for younger adults, whereas positive images compared to negative images activated this region more for older adults. The discrepancy between this study and the previous studies may be attributed to different experimental conditions (emotional autobiographical recall vs. processing of emotional images) and different baseline conditions. Another critical dissimilarity is that they showed below-baseline activation (i.e., deactivation) in the VMPFC/ACC region, while we found above-baseline activity in this region. The deactivation of the VMPFC/ACC might be due to the involvement of this region as a part of the DMN (Buckner et al., 2008) in their studies. However, the recall of emotional autobiographical memory used in the current study includes both self-referential processes and emotional processing that can induce the activation of the VMPFC/ACC. Thus, positive activity in this region was observed in the present study.

Interestingly, a significant interaction of age and valence on the activity patterns was observed in the left amygdala. The older adults produced stronger activation in the left amygdala during positive vs. negative recollection, while the younger adults showed the opposite effect. The present results are consistent with previous studies that revealed lower left amygdala activity during older adults' processing of negative information than positive information (Mather et al., 2004; Leclerc and Kensinger, 2010, 2011). The left-lateralized amygdala activity may reflect a more controlled, evaluative response than right amygdala activity (Phan et al., 2002; Costafreda et al., 2008). Therefore, older adults' reduced amygdala response to negative stimuli could be ascribed to their successful regulation of affective responses to negative stimuli. Moreover, many previous studies discovered that the age-related increase in the activity of the frontal cortex was always accompanied by diminished activation in the amygdala during the perception (Iidaka et al., 2002; St Jacques et al., 2010), as well as retrieval of negative stimuli (Murty et al., 2009). The amygdala's activity could be down-regulated by the VMPFC/ACC during emotional regulation and control processes (Ochsner et al., 2004; Urry et al., 2006; Delgado et al., 2008). Accordingly, our results of the VMPFC together with the left amygdala tended to support that the VMPFC/ACC played a role in emotional regulation, and possibly suggest that older adults' “positivity effect” may be associated with age-related changes in the VMPFC/ACC-amygdala circuit that plays an important role in emotional regulation. Because the VMPFC/ACC rather than the amygdala exhibited a significant correlation with valence intensity, the VMPFC/ACC could be a more critical region than the amygdala in its contribution to older adults' “positivity effect.”

### Limitations

Some aspects of the present experimental design limited the scope and interpretation of the corresponding data. Firstly, this study employed a semi-structured interview to elicit stimuli for presentation during scanning. A disadvantage of this method is that it may potentially change the essential nature of the memory we wish to examine. One possible solution to this problem is to glean the necessary information from spouses or family members of the subject as Viard et al. (2007) did. However, there is no guarantee that the subject could recall the specific events in a manner similar to their relatives. Moreover, emotional information that are collected from a third party rather than the subject himself/herself might not reflect true emotion and is likely to have equivocal emotional valence. Secondly, like many other studies investigating autobiographical event recollection, we used post-scanning debriefing to assess various retrieval qualities. As discussed by Oddo et al. (2010), the disadvantage of a post-scanning debriefing is that it is necessarily subjective. In future studies, additional physiological responses to emotional events should be recorded to guarantee more reliable measurements of emotion (Delgado et al., 2008). Thirdly, the two conditions (positive vs. negative retrieving) were split in two different runs. It is difficult to separate the effect of condition from the effect of run effect. The run effect may become a confounding factor that might influence the results to some extent. Finally, because only females and their recent memories were considered in this study, the current results cannot be generalized to males or to remote autobiographical memories.

## Conclusion

Taken together, our findings support the suggestion from previous studies of increased positive feelings in emotional autobiographical memory retrieval with advancing age. The imaging data indicated an age-related reversal within the VMPFC/ACC and amygdala of the brain networks that participated in the retrieval of emotional autobiographical events. Moreover, the activity in the VMPFC/ACC showed a negative correlation with the valence intensity. The results from this study may suggest that the positivity effect in older adults' autobiographical memories is associated with age-related changes in the controlled emotional processing implemented by the VMPFC/ACC-amygdala circuit.

### Conflict of interest statement

The authors declare that the research was conducted in the absence of any commercial or financial relationships that could be construed as a potential conflict of interest.

## Appendix

Five-point scales were used to specify the different aspects of recollective experience in terms of

1. Valence intensity for positive events retrieved in the scanner (1 = slightly positive to 5 = extremely positive);
2. Valence intensity for negative events retrieved in the scanner (1 = slightly negative to 5 = extremely negative);
3. Frequency of rehearsal (1 = never to 5 = very frequent);
4. Mental visual imagery quality/Vividness (1 = very blurry to 5 = very clear);
5. Personal significance (1 = not significant: it made no difference to my life and/or how I think about myself to 5 = great personal significance: it changed my life and/or how I view myself).
